# METTL1-mediated m7G modification regulates osteogenic differentiation of human periodontal ligament stem cells

**DOI:** 10.1016/j.clinsp.2025.100813

**Published:** 2025-10-28

**Authors:** Chungang Zhao, Kunlun Li, Aimin Wu

**Affiliations:** a School of Medicine, JingChu University of Technology, Jingmen, Hubei Province, China; b Jingmen Stomatological Hospital, Jingmen, Hubei Province, China

**Keywords:** Osteogenic differentiation, Periodontal ligament stem cells, METTL1

## Abstract

**Background:**

Human Periodontal Ligament Stem Cells (PDLSCs) represent a subset of mesenchymal stem cells originating from the periodontal ligament, a connective tissue responsible for anchoring teeth to alveolar bone. This study explored the functional role of Methyltransferase-Like-1 (METTL1)-mediated N7-methylguanosine (m7G) modification in regulating the osteogenic differentiation capacity of PDLSCs.

**Methods:**

PDLSCs were isolated from periodontal ligament tissues. Osteogenic differentiation was assessed through Alkaline Phosphatase (ALP) activity assays and Alizarin Red S (ARS) staining. Expression levels of Runt-related Transcription Factor-2 (RUNX2), Osteocalcin (OCN), and Osteopontin (OPN) were quantified using Real-Time quantitative Polymerase Chain Reaction (RT-qPCR). Western blot analysis was employed to detect protein expression of METTL1, NOP2/Sun RNA Methyltransferase-2 (NSUN2), and WD Repeat Domain-4 (WDR4). Total m7G content in PDLSCs was measured via m7G dot blot assays. The interaction between METTL1 and RUNX2 was validated using luciferase reporter assays.

**Results:**

The present findings demonstrated that METTL1 exhibited elevated expression levels in PDLSCs, with further upregulation during osteogenic induction. METTL1 knockdown significantly impaired osteogenic differentiation, characterized by decreased ALP activity, reduced ARS staining intensity, and downregulated osteogenic marker gene expression. Mechanistically, the authors identified that METTL1 enhanced m7G modification of key osteogenic genes, including RUNX2, OCN, and OPN, thereby improving their mRNA stability and translational efficiency. Notably, forced overexpression of RUNX2 partially reversed the osteogenic differentiation defects induced by METTL1 suppression.

**Conclusion:**

METTL1 promotes osteogenic differentiation in PDLSCs through m7G modification-mediated stabilization of RUNX2 expression. This discovery unveils a novel epigenetic regulatory mechanism involving m7G modification in periodontal tissue regeneration, offering potential therapeutic targets for bone defect repair applications.

## Highlights

• METTL1 expression increases in PDLSCs during osteogenic differentiation.
• METTL1 mediates RUNX2 mRNA stability in PDLSCs through m7G modification.
• High METTL1 boosts PDLSC osteogenesis through m7G-dependent RUNX2 activation.

## Introduction

Osteogenic differentiation is a biological process involving the commitment of Mesenchymal Stem Cells (MSCs) to osteoblasts, which play a critical role in bone formation and remodeling.^1^ This differentiation process is regulated by multiple factors, including transcription factors, growth factors, signaling pathways, and epigenetic modifications.^1^, ^2^, ^3^ Elucidating the molecular mechanisms underlying osteogenic differentiation is crucial for designing innovative therapeutic approaches to bone regeneration and tissue repair.

Human Periodontal Ligament Stem Cells (PDLSCs) represent a subset of MSCs derived from the periodontal ligament, a connective tissue responsible for anchoring teeth to alveolar bone.^4^ These cells exhibit self-renewal and multipotent differentiation capabilities, along with immunomodulatory and anti-inflammatory properties.^5^^,^^6^ Due to their clinical advantages ‒ including easy accessibility, rapid proliferation rates, and exceptional osteogenic potential ‒ PDLSCs have become widely utilized as seed cells for tissue engineering and regenerative medicine applications.^7^ Furthermore, PDLSCs serve as key progenitor cells for periodontal regeneration, owing to their multipotency, self-renewal capacity, and especially their robust osteogenic potential. Their ability to promote bone formation and seamlessly integrate with native periodontal structures highlights their significant therapeutic value in regenerative dentistry.^8^^,^^9^

N7-methylguanosine (m7G) modification is a post-transcriptional RNA modification occurring at the N7 position of guanosine residues in RNA molecules, including tRNAs, rRNAs, mRNAs, and miRNAs.^10^ This modification is catalyzed by a family of methyltransferases, with Methyltransferase-Like-1 (METTL1) serving as the principal enzyme responsible for m7G modification in tRNAs and mRNAs.^11^^,^^12^ Accumulating evidence suggests that m7G modification plays critical roles in multiple aspects of RNA metabolism, including splicing, nuclear export, stability, and translational efficiency.^13^^,^^14^ Recent studies have highlighted the regulatory significance of m7G modification in stem cell biology, particularly in maintaining pluripotency and directing differentiation processes. For example, METTL1-mediated m7G modification of tRNAs and mRNAs has been shown to sustain the self-renewal of mouse embryonic stem cells and human induced pluripotent stem cells while restricting their differentiation into mesodermal and vascular lineages.^15^, ^16^, ^17^ However, the functional role of METTL1 and its associated m7G modification in the osteogenic differentiation of PDLSCs remains poorly characterized. Given the clinical relevance of PDLSCs in applications such as bone repair and periodontal regeneration, elucidating how METTL1-mediated m7G modification regulates their osteogenic capacity could yield novel therapeutic strategies for periodontal diseases.^18^^,^^19^ Moreover, uncovering this mechanism would contribute to the broader understanding of RNA modifications in stem cell biology, bridging molecular regulatory pathways with tissue-specific regenerative outcomes.^20^

In this study, the authors hypothesized that METTL1-mediated m7G modification promotes the osteogenic differentiation of PDLSCs. To validate this hypothesis, the authors first assessed the expression profile and functional role of METTL1 in PDLSCs under osteogenic induction conditions. Subsequently, the authors investigated the impact of METTL1 on the m7G modification status of osteogenesis-related genes, as well as their mRNA expression and translational efficiency. Finally, the authors conducted a rescue experiment to evaluate the contribution of RUNX2, a pivotal transcription factor in osteogenesis, to the METTL1-regulated osteogenic differentiation of PDLSCs.

## Materials and methods

### Isolation and culture of PDLSCs

PDLSCs were isolated from the periodontal ligament of healthy third molars extracted from patients aged 18–25 years under written informed consent. The study was approved by the Ethics Committee of the JingChu University of Technology (approval number: LL2521). Periodontal ligament tissue was minced and digested with 3 mg/mL type I collagenase and 4 mg/mL dispase for 1 hour at 37°C. The resulting single-cell suspension was seeded into 25 cm^2^ culture flasks and cultured in Dulbecco’s Modified Eagle’s Medium (DMEM) supplemented with 10% Fetal Bovine Serum (FBS), 100 U/mL penicillin, and 100 μg/mL streptomycin in a humidified incubator at 37°C with 5% CO_2_. The medium was replaced every 3-days, and cells were passaged upon reaching 80%–90% confluence.

### Osteogenic induction

PDLSCs at passages 3–5 were used for osteogenic induction. The cells were seeded in 6-well plates at a density of 1×10^4^ cells/cm^2^ and cultured in Osteogenic induction Medium (OM), comprising Dulbecco’s Modified Eagle’s Medium (DMEM) supplemented with the following: 10% FBS, 100 U/mL penicillin, 100 μg/mL streptomycin, 10 mM β-glycerophosphate, 50 μg/mL ascorbic acid, and 100 nM dexamethasone. The medium was replaced every 3-days. Cells were harvested on days 0, 4, 7, and 14 for subsequent analysis.

### Immunofluorescence staining

To assess the osteogenic differentiation of PDLSCs, Alkaline Phosphatase (ALP) and Alizarin Red-S (ARS) staining were performed at designated time points. For ALP staining, cells were fixed with 4% paraformaldehyde for 15-minutes and stained using an ALP staining kit (Beyotime, China) following the manufacturer’s instructions. For ARS staining, cells were fixed with 70% ethanol for 1 hour and stained with 2% ARS solution (pH 4.2) for 10 minutes. Stained cells were visualized and imaged under a light microscope. Quantification of ALP and ARS staining was achieved by extracting the dye with 10% cetylpyridinium chloride and measuring absorbance at 520 nm and 405 nm, respectively.

### Real-time polymerase chain reaction (PCR)

Total RNA was extracted from PDLSCs using TRIzol reagent (Invitrogen, USA) and reverse-transcribed into cDNA using a PrimeScript RT reagent kit (Takara, Japan). Quantitative real-time PCR (qRT-PCR) was performed on a CFX96 Touch Real-Time PCR Detection System (Bio-Rad, USA) using a SYBR Premix Ex Taq kit (Takara, Japan). The relative expression levels of target genes were normalized to GAPDH and calculated using the 2^−ΔΔCT^ method.

### Dot blot assay

To assess the contribution of m7G modification to the osteogenic differentiation of PDLSCs, an m7G dot blot assay was performed to quantify global m7G modification levels in the cells at designated time points. Briefly, 2 μg of total RNA was spotted onto a nitrocellulose membrane and cross-linked using UV irradiation. The membrane was blocked with 5% non-fat milk in TBST for 1 h and incubated with anti-m7G antibody (1:1000 dilution; Abcam, UK) overnight at 4°C. After washing with TBST, the membrane was incubated with HRP-conjugated secondary antibody (1:5000 dilution; Beyotime, China) for 1 hour at room temperature. The signal was visualized using Enhanced Chemiluminescence (ECL) and analyzed with ImageJ software.

### Western-blot analysis

PDLSCs cultured under osteogenic conditions for 14 days were lysed using radioimmunoprecipitation assay buffer (Beyotime, China) supplemented with a protease inhibitor cocktail (Roche, Switzerland). Protein concentrations were quantified using the bicinchoninic acid assay (Beyotime, China). Equal amounts of protein (20 μg) were resolved by sodium dodecyl sulfate-polyacrylamide gel electrophoresis and transferred onto a polyvinylidene fluoride membrane. The membrane was blocked with 5% non-fat milk in TBST for 1 hour and incubated overnight at 4°C with primary antibodies against METTL1 (1:1000 dilution; Abcam, UK), METTL14 (1:1000 dilution; Abcam, UK), METTL16 (1:1000 dilution; Abcam, UK), WTAP (1:1000 dilution; Abcam, UK), ALKBH5 (1:1000 dilution; Abcam, UK), and GAPDH (1:5000 dilution; Beyotime, China). After washing with TBST, the membrane was incubated with HRP-conjugated secondary antibody (1:5000 dilution; Beyotime, China) for 1 hour at room temperature. The signal was detected using ECL and quantified with ImageJ software. The expression levels of m7G-related enzymes were further visualized as a heatmap generated using R software.

### METTL1 knockdown

To investigate the functional role of METTL1 in the osteogenic differentiation of PDLSCs, METTL1 was knocked down using shRNA-mediated gene silencing. The cells were transfected with shMETTL1 or shNC (negative control) plasmids (GenePharma, China) using Lipofectamine 3000 reagent (Invitrogen, USA) according to the manufacturer’s instructions. The knockdown efficiency was confirmed by qRT-PCR and Western blotting.

### Luciferase reporter assay

To further validate the role of METTL1 in the m7G modification of RUNX2, the authors engineered three luciferase reporter plasmids containing the 5′UTR of RUNX2 mRNA, which harbors predicted m7G modification sites at positions 241–480 bp, 721–960 bp, and 2161–2400 bp, respectively (Fig. 4B). The cells were co-transfected with wild-type or mutant reporter plasmids and a Renilla luciferase plasmid (as an internal control) using Lipofectamine 3000 reagent (Invitrogen, USA) according to the manufacturer’s instructions. Luciferase activity was measured 48 hours post-transfection using the Dual-Luciferase Reporter Assay System (Promega, USA). Relative luciferase activity was calculated by normalizing Firefly luciferase activity to Renilla luciferase activity.

### Rescue experiment

To determine whether RUNX2 overexpression could mitigate the osteogenic differentiation impairment induced by METTL1 knockdown, the cells were co-transfected with shMETTL1 and either a RUNX2 overexpression plasmid (GenePharma, China) or an empty vector (control). The efficacy of RUNX2 overexpression was confirmed by qRT-PCR and Western blotting. The transfected cells were cultured in OM for 14-days and subsequently analyzed for ALP and ARS staining, as well as osteogenic marker gene expression, following the protocols outlined above

## Results

### Osteogenic induction of PDLSCs

**Fig. 1:**
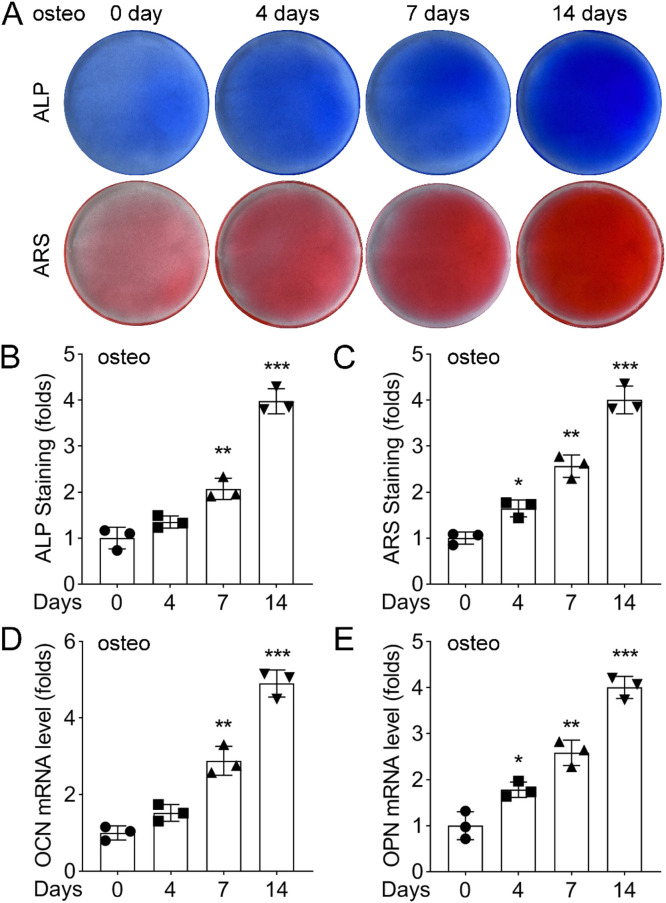
Phenotypic changes of PDLSCs under osteogenic induction. (A) ALP staining of PDLSCs cultured in normal or osteogenic medium for 0, 4, 7, and 14 days. The color changed from light blue to dark blue, indicating the increase of ALP activity. (B) ARS staining of PDLSCs cultured in normal or osteogenic medium for 0, 4, 7, and 14 days. The color changed from light red to dark red, indicating the increase of calcium deposition. (C) Quantification of ALP activity by measuring the absorbance at 562 nm. (D) Quantification of calcium deposition by measuring the absorbance at 405 nm. Data are presented as mean ± SD of three independent experiments. ** p < 0.01, *** p < 0.001, compared with the normal medium group at the same time point.

To observe changes in ALP activity and mineralization during osteogenic induction, the authors conducted ARS staining at 0, 4, 7, and 14 days. Results demonstrated that the color of ALP staining (upper panel) transitioned from light blue to dark blue, indicating an increase in ALP activity. Similarly, the color of ARS staining (lower panel) shifted from light red to dark red, reflecting enhanced mineralization (Fig. 1A‒C). Furthermore, the expression levels of OCN and OPN in PDLSCs were significantly upregulated at 4, 7, and 14 days after osteogenic induction (Fig. 1D and E). These findings indicate that osteogenic markers progressively increased with prolonged osteogenic induction.

### m7G-related enzyme of METTL1 was upregulated during osteogenic differentiation of PDLSCs

**Fig. 2:**
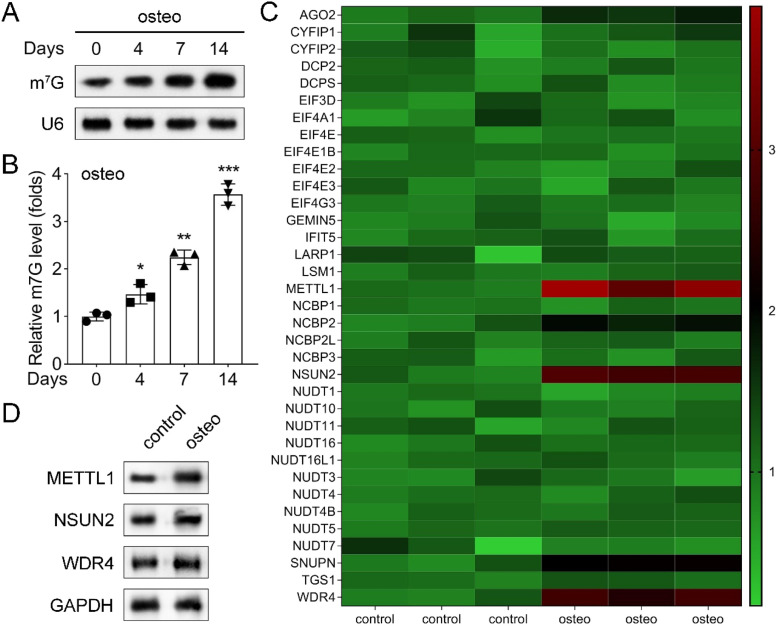
m7G modification changes during osteogenic differentiation. (A) m7G dot blot assay of total RNA extracted from PDLSCs cultured in normal or osteogenic medium for 0, 4, 7, and 14 days. The intensity of the dots indicated the level of m7G modification. (B) Quantification of m7G level by ImageJ software. (C) Heatmap of the expression of m7G-related enzymes (METTL1, METTL3, METTL14, METTL16, ALKBH5, and FTO) in PDLSCs cultured in normal or osteogenic medium for 14-days. The color scale represents the relative expression level. (D) Western blot analysis of the expression of m7G-related enzymes in PDLSCs cultured in normal or osteogenic medium for 14-days. GAPDH was used as a loading control. Data are presented as mean ± SD of three independent experiments. * p < 0.05, ** p < 0.01, compared with the normal medium group.

To evaluate the global level of m7G modification during osteogenic differentiation, the authors performed an m7G dot blot assay at designated time points. The m7G level increased progressively during osteogenic induction (Fig. 2A), as quantified by the corresponding analysis of m7G abundance (Fig. 2B).

To identify m7G-related enzymes associated with the osteogenic differentiation of PDLSCs, the authors analyzed the expression of METTL1, METTL14, METTL16, WTAP, and ALKBH5 via Western blotting in control and 14-day osteogenic-induced cells. METTL1 exhibited the highest expression among these enzymes in both groups and was significantly upregulated following osteogenic induction. The expression levels of the remaining enzymes remained largely unchanged or exhibited only minor fluctuations in response to osteogenic induction. The heatmap further highlighted the differential expression patterns of these enzymes between the two groups (Fig. 2C and 2D).

### METTL1 knockdown impaired osteogenic differentiation of PDLSCs

**Fig. 3:**
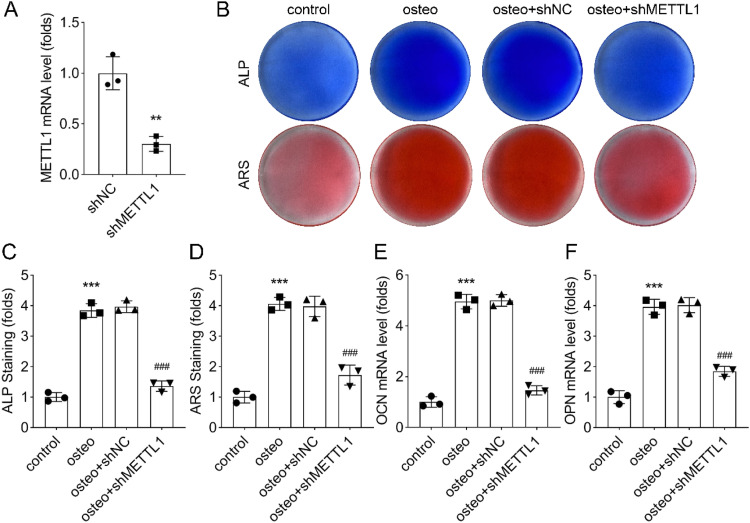
Effect of METTL1 on osteogenic differentiation. (A) qRT-PCR and western blot analysis of METTL1 expression in PDLSCs infected with shNC or shMETTL1. GAPDH was used as a loading control. (B) ALP and ARS staining of PDLSCs infected with shNC or shMETTL1 and cultured in normal or osteogenic medium for 14-days. (C‒F) Quantification of ALP activity, calcium deposition, and expression of osteogenic marker genes (ALP, OCN, and OPN) by qRT-PCR. Data are presented as mean ± SD of three independent experiments. * p < 0.05, ** p < 0.01, *** p < 0.001, compared with the shNC group under the same culture condition.

To investigate the functional role of METTL1 in the osteogenic differentiation of PDLSCs, the authors performed shRNA-mediated METTL1 knockdown. The knockdown efficiency was validated by qRT-PCR (Fig. 3A). Subsequently, the authors compared osteogenic differentiation in the control, OI, OI+shNC, and OI+shMETTL1 groups. ARS staining revealed significantly reduced alkaline phosphatase activity and mineralization in the OI+shMETTL1 group compared to the OI+shNC group (Fig. 3B‒D). Statistical analysis further demonstrated that METTL1 knockdown markedly decreased the expression levels of osteogenic marker genes OCN and OPN (Fig. 3E‒F). These findings suggest that METTL1 knockdown impairs the osteogenic differentiation capacity of PDLSCs.

### METTL1 enhanced the translation of osteogenic-related RUNX2

**Fig. 4:**
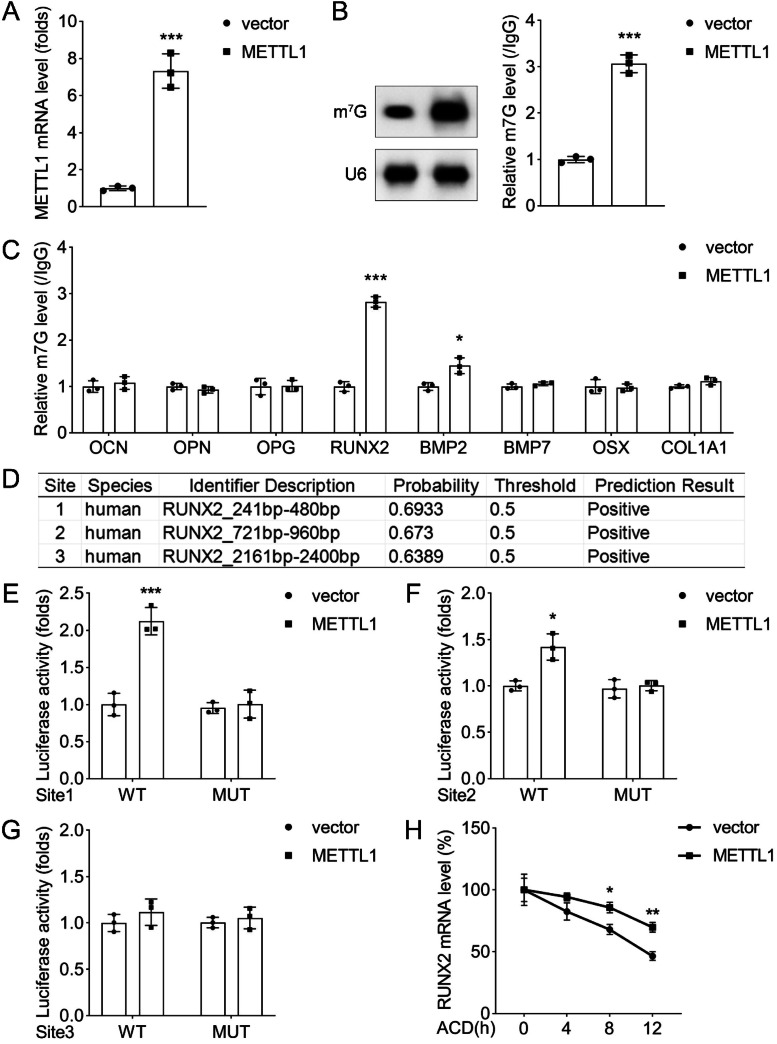
Evidence of METTL1-mediated regulation of osteogenic-related genes. (A) METTL1 overexpression plasmids transfection efficiency was investigated by qPCR. (B) The m7G levels in PDLSCs were detected by western blot. (C) The 7G-modified RNA of osteogenic-related genes (OCN, OPN, OPG, RUNX2, BMP2, BMP7, Osx, and COL1A1) in PDLSCs infected with control vector or METTL1 plasmid and cultured in osteogenic medium for 14-days. The enrichment of m7G-modified RNA was calculated by the percentage of input method. (D) The potential m7G modification sites of METTL1 in the 5’UTRs of RUNX2 were constructed. (E‒G) Luciferase reporter assay of the RUNX2 expression in PDLSCs infected with METTL1 plasmid combined with the plasmids of each binding sites on RUNX2, and cultured in osteogenic medium for 14-days. The luciferase activity was normalized to the Renilla luciferase activity. (H) ACD assay of AID-tagged METTL1 and its catalytically inactive mutant (METTL1-D304A) in PDLSCs transfected with the luciferase reporter vectors containing the 5’UTRs of RNX2. The cells were treated with 500 μM auxin for 24h to induce the degradation of AID-tagged METTL1 proteins. The luciferase activity was normalized to the Renilla luciferase activity. Data are presented as mean ± SD of three independent experiments. * p < 0.05, ** p < 0.01, *** p < 0.001, compared with the corresponding control group.

Subsequently, the authors investigated the role of METTL1 on m7G modification in PDLSCs. qPCR suggested that METTL1 expression was elevated following METTL1 overexpression (Fig. 4A). Moreover, METTL1 overexpression increased the m7G level in PDLSCs (Fig. 4B). To elucidate how METTL1 regulates the expression of osteogenic-related genes, the authors performed meRIP assays to analyze m7G modification levels in the control and OI+shMETTL1 groups. The m7G enrichment of RUNX2, OCN, and OPN was significantly increased by METTL1 overexpression (Fig. 4C), indicating that METTL1 enhances m7G modification of these transcripts.

To further validate the role of METTL1 in RUNX2 m7G modification, the authors constructed three luciferase reporter plasmids containing the 5′UTR of RUNX2 mRNA, which harbored putative m7G sites at positions 241–480 bp, 721–960 bp, and 2161–2400 bp, respectively (Fig. 4D). Luciferase activity of the wild-type plasmid was markedly higher than that of the mutant plasmid, indicating that all three m7G sites were functionally important for RUNX2 mRNA translation. Moreover, luciferase activity of the wild-type plasmid was significantly elevated by METTL1 overexpression, whereas that of the mutant plasmid remained unchanged (Fig. 4E‒G), demonstrating that METTL1 specifically enhances m7G modification and subsequent translation of RUNX2 mRNA (Fig. 4H).

### RUNX2 could rescue the osteogenic defect caused by METTL1 knockdown

**Fig. 5:**
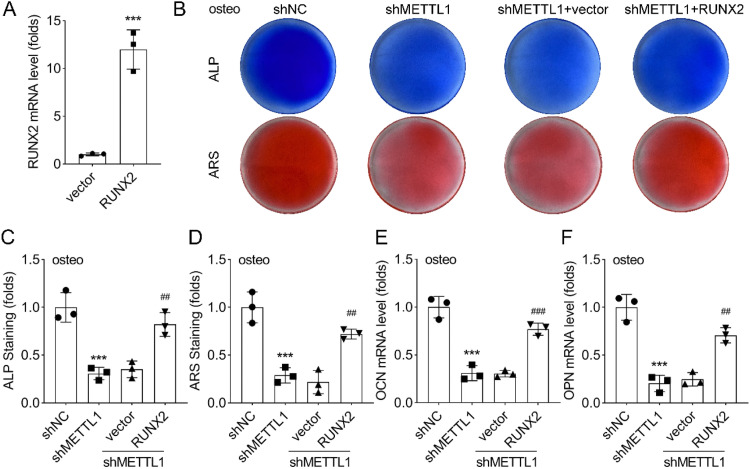
Rescue experiment of METTL1 function. (A) qRT-PCR and western blot analysis of RUNX2 expression in PDLSCs co-infected with shNC or shMETTL1 and RUNX2 overexpression vector or empty vector. GAPDH was used as a loading control. (B‒D) Quantification of ALP and ARS staining of PDLSCs co-infected with shNC or shMETTL1 and RUNX2 overexpression vector or empty vector and cultured in normal or osteogenic medium for 14-days. (E‒F) Quantification of expression of osteogenic marker genes (OCN and OPN) by qRT-PCR. Data are presented as mean ± SD of three independent experiments. * p < 0.05, ** p < 0.01, *** p < 0.001, compared with the corresponding control group.

To determine whether RUNX2 overexpression could reverse the osteogenic differentiation impairment induced by METTL1 knockdown, the cells were co-transfected with shMETTL1 and either a RUNX2 overexpression plasmid or a control vector. The efficacy of RUNX2 overexpression was validated by qRT-PCR (Fig. 5A). As illustrated in Fig. 5B, ALP activity and mineralization were partially restored in METTL1-knockdown cells following RUNX2 overexpression. Statistical analysis (Fig. 5C‒F) demonstrated that the expression levels of osteogenic marker genes (ALP, OCN, and OPN) were also partially reversed by RUNX2 overexpression. These findings suggest that RUNX2 overexpression mitigates, at least in part, the osteogenic differentiation defect caused by METTL1 knockdown.

## Discussion

In this study, the authors investigated the role of METTL1-mediated m7G modification in the osteogenic differentiation of PDLSCs. The authors observed that METTL1 was significantly upregulated during osteogenic induction, and its knockdown impaired the osteogenic differentiation capacity of PDLSCs. Furthermore, the present data demonstrated that METTL1 enhances m7G modification of osteogenic-related genes, thereby increasing their mRNA stability and translation efficiency. Conversely, overexpression of RUNX2 partially reversed the osteogenic differentiation defect caused by METTL1 knockdown. These findings highlight the critical role of METTL1-mediated m7G modification in regulating osteogenic differentiation and suggest that targeting the METTL1-RUNX2 axis may represent a promising therapeutic strategy for tissue engineering applications.

Our findings are consistent with previous studies demonstrating that m7G modification plays a pivotal role in regulating stem cell fate and differentiation.^21^ For example, METTL1 has been shown to enhance the self-renewal and pluripotency of embryonic stem cells by promoting m7G modification and translation of NANOG mRNA.^22^ Similarly, METTL1 facilitates adipogenic differentiation of MSCs by upregulating m7G modification and expression of PPARγ, a master regulator of adipogenesis.^23^ Additionally, METTL14, another m7G methyltransferase, modulates neuronal differentiation of neural stem cells through regulation of m7G modification and splicing of SOX2 mRNA.^24^ Collectively, these studies, along with these results, highlight that m7G modification is a versatile and dynamic epitranscriptomic regulatory mechanism that governs the expression of critical genes driving stem cell differentiation.^25^

Among the m7G-related enzymes, the authors observed that METTL1 was the most highly expressed and significantly upregulated in PDLSCs during osteogenic induction. This suggests that METTL1 is the primary enzyme mediating m7G modification in PDLSCs. However, the authors cannot rule out the possibility that other enzymes, such as METTL14, METTL16, WTAP, and ALKBH5, may also contribute to m7G modification in PDLSCs, albeit to a lesser extent. Future studies are warranted to further elucidate the functional interactions and potential crosstalk among these enzymes in the regulation of PDLSC differentiation.

RUNX2 is a pivotal transcription factor for osteogenesis, as it activates the expression of key osteogenic genes, including OCN, OPN, and OPG.^26^ The authors demonstrated that METTL1 functions as a positive regulator of RUNX2 expression and activity. Conversely, METTL1 knockdown reduced m7G modification and translation of RUNX2 mRNA, leading to decreased RUNX2 protein levels and impaired osteogenic differentiation. Interestingly, RUNX2 overexpression partially rescued the osteogenic defect caused by METTL1 deficiency, indicating that RUNX2 acts as a downstream effector of METTL1-mediated osteogenic differentiation. These findings align with a recent study by Liu et al.^27^ who reported that METTL1 expression was significantly reduced in Bone Marrow Mesenchymal Stem Cells (BMSCs) from patients with Senile Osteoporosis (SOP). METTL1 deficiency in BMSCs markedly inhibited m7G modification of RUNX2, suppressed osteogenic differentiation *in vitro*, and induced SOP *in vivo*. Notably, lower osteogenic differentiation capacity in BMSCs correlated with reduced METTL1 expression, and METTL1 deficiency further impaired osteogenesis by decreasing RUNX2 m7G modification and downregulating its expression. Compared to these findings, the present results confirm the critical role of the METTL1/m7G/RUNX2 axis in PDLSC osteogenic differentiation, offering a novel theoretical framework for leveraging PDLSCs in periodontal tissue regeneration. Furthermore, the incomplete rescue of osteogenic defects by RUNX2 overexpression suggests that METTL1 may regulate additional osteogenic genes beyond RUNX2.^28^ Indeed, the authors observed that METTL1 enhances m7G modification and expression of multiple osteogenic genes, including OCN, OPN, OPG, BMP2, BMP7, Osx, and COL1A1. These genes orchestrate diverse aspects of osteogenesis, such as extracellular matrix synthesis, mineralization, signaling pathways, and transcriptional regulation.^29^, ^30^, ^31^ Therefore, METTL1 likely exerts a comprehensive and coordinated regulatory effect on PDLSC osteogenic differentiation by modulating the expression of multiple osteogenic genes through m7G modification.

This study has several limitations that warrant consideration. First, these findings are based exclusively on *in vitro* experiments, and the role of the METTL1/RUNX2 axis in PDLSC osteogenic differentiation has not yet been validated in *in vivo* models or clinical settings. While the mechanistic insights provided by this study are valuable, further investigations are necessary to confirm these findings in animal models of periodontal regeneration and translational clinical studies. Additionally, future research should explore potential crosstalk between METTL1 and other regulatory pathways in PDLSCs, as well as the long-term functional outcomes of METTL1 modulation in tissue engineering applications.

## Conclusion

In summary, the present findings demonstrate that METTL1-mediated m7G modification is a critical regulatory mechanism underlying the osteogenic differentiation of PDLSCs. These results uncover a novel epitranscriptomic mechanism governing PDLSC osteogenesis and identify METTL1 as a potential therapeutic target in tissue engineering applications.

## Authors’ contributions

All authors contributed to the study conception and design. Material preparation, data collection and analysis were performed by Chungang Zhao, Kunlun Li and Aimin Wu. The first draft of the manuscript was written by Chungang Zhao and all authors commented on previous versions of the manuscript. All authors read and approved the final manuscript.

## Funding

This studuy was supported by Guiding Project of Hubei Provincial Department of Education's Scientific Research Plan (No. B2024211), JingChu University of Technology Full Life Cycle Oral Health Promotion and Communication Research Team.

## Ethical approval

The study was approved by the Ethics Committee of the JingChu University of Technology (approval number: LL2521). Written informed consent was obtained from all patients. All experiments were performed in accordance with relevant guidelines and regulations.

## Data availability statements

The datasets used and/or analyzed during the current study are available from the corresponding author on reasonable request.

## Declaration of competing interest

The authors declare no conflicts of interest.
